# Unloading‐Induced Skeletal Interoception Alters Hypothalamic Signaling to Promote Bone Loss and Fat Metabolism

**DOI:** 10.1002/advs.202305042

**Published:** 2023-10-25

**Authors:** Qiaoyue Guo, Ningrong Chen, Kalp Patel, Mei Wan, Junying Zheng, Xu Cao

**Affiliations:** ^1^ Department of Orthopedic Surgery Johns Hopkins University School of Medicine Baltimore MD 21205 USA; ^2^ Department of Endocrinology Endocrinology Research Center Xiangya Hospital of Central South University Changsha Hunan 410008 China; ^3^ Department of Biomedical Engineering Johns Hopkins University School of Medicine Baltimore MD 21205 USA

**Keywords:** bone metabolism, microgravity, neuropeptide Y (NPY), skeletal interoception, tyrosine hydroxylase (TH)

## Abstract

Microgravity is the primary factor that affects human physiology in spaceflight, particularly bone loss and disturbances of the central nervous system. However, little is known about the cellular and molecular mechanisms of these effects. Here, it is reported that in mice hindlimb unloading stimulates expression of neuropeptide Y (NPY) and tyrosine hydroxylase (TH) in the hypothalamus, resulting in bone loss and altered fat metabolism. Enhanced expression of TH and NPY in the hypothalamus occurs downstream of a reduced prostaglandin E2 (PGE2)‐mediated ascending interoceptive signaling of the skeletal interoception. Sympathetic antagonist propranolol or deletion of *Adrb2* in osteocytes rescue bone loss in the unloading model. Moreover, depletion of TH^+^ sympathetic nerves or inhibition of norepinephrine release ameliorated bone resorption. Stereotactic inhibition of NPY expression in the hypothalamic neurons reduces the food intake with altered energy expenditure with a limited effect on bone, indicating hypothalamic neuroendocrine factor NPY in the facilitation of bone formation by sympathetic TH activity. These findings suggest that reduced PGE2‐mediated interoceptive signaling in response to microgravity or unloading has impacts on the skeletal and central nervous systems that are reciprocally regulated.

## Graphical abstract

During unloading, PGE2/EP4 ascending signal increases the expression of both sympathetic tone and NPY level in the hypothalamus. The elevated NE leads to bone loss while increased hypothalamic NPY acts as neuronal endocrine factor for the impaired metabolism.  The primary function of NPY is to regulate energy metabolism to facilitate bone formation.

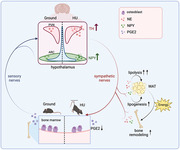

## 1 Introduction

The potential capacity for manned spaceflight, including for commercial ventures, has rapidly developed. As the duration of human space travel would increase in such instances, it is important to understand how spaceflight affects physiology and health under microgravity.^[^ ^1^, ^2^ ^]^ Indeed, currently one of the major drawbacks against spaceflight is the experience of microgravity, which has profound effects on the musculoskeletal system including bone loss, muscle atrophy, and negative effects on the cardiovascular and immune systems.^[^ ^3^, ^4^, ^5^, ^6^ ^]^ Particularly, long‐duration space travel has detrimental effects on the human central nervous system (CNS),^[^ ^7^, ^8^ ^]^ including those involving neurovestibular function, as well as psychological disturbances and cognitive changes.^[^ ^9^, ^10^ ^]^ Likewise, animal studies have shown that microgravity alters neuroplasticity.^[^ ^11^ ^]^ However, the cellular and molecular mechanisms for the effects of microgravity on CNS are unclear.

Terrestrial life on Earth evolved within a relatively narrow range of gravitational forces. In particular, the skeletal system evolved to support and protect the internal organs, as well as allowing for locomotion, but also to act as an important mineral reserve.^[^ ^12^, ^13^ ^]^ As such, the bone is a mechanosensitive tissue and mechanical loading is essential for its homeostasis.^[^ ^14^, ^15^ ^]^ Under microgravity, humans in spaceflight lose 1 to 3 percent of their bone mass each month.^[^ ^16^, ^17^ ^]^ It is unclear under such conditions if bone loss is associated with alterations in the CNS. We recently uncovered that the signals induced by mechanical loading on the bone are processed by the brain to regulate bone formation and metabolism through a process called skeletal interoception.^[^ ^18^, ^19^, ^20^, ^21^ ^]^ The discovery of skeletal interoception provides a unique opportunity to investigate how microgravity affects CNS function.

Interoception in general refers to an organism's ability to sense its internal state, and this sensation relies on a complex series of interactions between the brain and peripheral organs to maintain homeostasis.^[^ ^20^, ^22^ ^]^ Skeletal interoception, more specifically, involves the ability of the brain to recognize changes in the bone, for example in response to mechanical loading or to low bone density.^[^ ^20^ ^]^ We have recently reported that under such conditions PGE2 is secreted by osteoblasts, which activates PGE2 receptor 4 (EP4) in ascending sensory nerves along the interoceptive pathway. This activation promotes cAMP response element‐binding (CREB) signaling in the hypothalamus to elevate tyrosine hydroxylase (TH) expression that regulates sympathetic activity and bone metabolism.^[^ ^18^, ^21^ ^]^ Interestingly, studies on space flights have shown that different types of cultured osteoblastic cells show either a reduction or an increase or no change in PGE2 expression,^[^ ^23^, ^24^, ^25^ ^]^ but the effect on PGE2 levels in vivo is still unclear. Therefore, if the PGE2 levels are decreased under the microgravity conditions that occur during spaceflight, that could alter the hypothalamic function and thus explain the resulting bone loss observed under such conditions.

The hindlimb unloading (HU) model was developed in the 1980s to simulate microgravity on Earth.^[^ ^26^, ^27^ ^]^ The results of the HU of experiments were consistent with the observation of the musculoskeletal system sensitive to microgravity in early spaceflight.^[^ ^27^, ^28^ ^]^ Since its development, the HU model has been used extensively to investigate the influence of an unloading environment as an analog of spaceflight‐induced weightlessness on integrated physiology, organ‐specific effects, and mechanistic responses.^[^ ^26^, ^29^ ^]^ Significant progress has been made using the HU microgravity analog model to understand the functional changes of osteoclasts and osteoblasts in bone loss.^[^ ^30^, ^31^ ^]^ In addition to a well‐characterized simulation of being in space on the musculoskeletal system, the HU model has been used successfully as an analog of microgravity. Importantly, PGE2 levels in the bone marrow are lower upon mechanical unloading,^[^ ^21^ ^]^ a condition that is associated with bone loss. In contrast, mechanical loading leads to increased PGE2 secretion by osteoblasts, which induces activation of CREB phosphorylation in the hypothalamus and the downregulation of sympathetic outflow, leading to increased bone mass accrual by stimulating osteogenic differentiation of bone marrow mesenchymal stem/stromal cells (BMSCs).^[^ ^18^, ^21^ ^]^ We also found that elevated sympathetic activity leads to both intra‐cortical bone and trabecular bone remodeling in lactating mice.^[^ ^32^ ^]^ Thus, it is possible that the hypothalamus perceives the downregulated PGE2 skeletal interoceptive signaling to regulate bone architecture during unloading or microgravity. PGE2 skeletal interoceptive signaling may also regulate hypothalamic NPY expression to induce WAT lipolysis to provide energy for osteoblastic bone formation as a parallel descending neuroendocrine pathway.^[^ ^19^ ^]^ Therefore, the decreased bone marrow PGE2 levels in the HU model could significantly increase hypothalamic NPY expression, leading to altered metabolism between bone and fat.

In the current study, we used the HU model to investigate whether changes in skeletal interoception alter hypothalamic activity to regulate the skeletal system. In this way, we found that skeletal interoception and its regulation of bone density is mediated by alterations in hypothalamic function due to changes in skeletal PGE2 levels. Notably, we found that decreased PGE2/EP4‐mediated interoceptive ascending signaling stimulates both TH and NPY expression in the hypothalamus after two weeks of unloading. These changes in TH levels resulted in elevated sympathetic tone and induction of osteocyte‐mediated bone resorption and WAT lipolysis, while the elevated NPY levels altered energy expenditure and metabolism. Moreover, inhibition of sympathetic output reduced bone loss and fat metabolism in the unloaded mice, whereas inhibition of hypothalamic NPY expression only accelerated fat lipolysis with limited effect on bone loss. Our findings for the first time demonstrate that weightlessness affects brain function to regulate bone remodeling and metabolism via PGE2/EP4‐driven skeletal interoception.

## 2 Results

### 2.1 Unloading Leads to Reduced PGE2‐Mediated Interoceptive Signaling and Enhanced Sympathetic TH Expression

**Figure 1:**
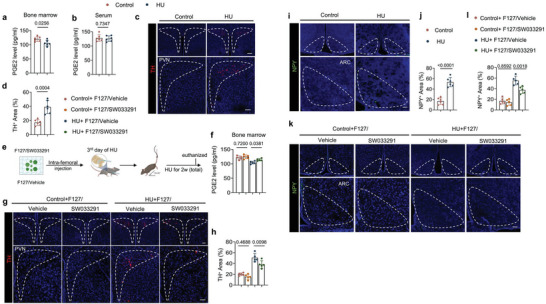
PGE2 ascending interoceptive signaling affects both hypothalamic tyrosine hydroxylase (TH) expression and neuropeptide Y (NPY) expression in HU mice. a,b) Enzyme‐linked immunosorbent assay (ELISA) analysis of PGE2 level in bone marrow (a) and serum (b) of control and HU mice. c) Representative immunofluorescent staining of TH‐positive nerve neurons and fibers (red) from the paraventricular nucleus (PVN) of the hypothalamus and d) their quantitative analysis in control and HU mice. Higher magnifications of selected areas are shown at the lower side of corresponding panels. Scale bar = 40 µm. *N* = 6. e) Schematic diagram illustrating the procedure of bone marrow injection of F127/vehicle (30% w/v F127 in PBS) and F127/SW033219 (15 mg mL^−1^ of SW033219 dissolved in 30% w/v F127 of PBS, the dose of SW033219 was 120 mg kg^−1^) in HU mice. Mice were given intra‐bone marrow injections on the 3rd day of unloading. f) PGE2 level in bone marrow determined by ELISA analysis of control and HU mice treated with F127/vehicle and F127/SW033219, respectively. *N* = 5. g) Representative immunofluorescent staining of TH‐positive nerve neurons and fibers (red) from PVN of hypothalamus and h) their quantitative analysis in different treatment groups. Higher magnifications of selected areas are shown at the lower side of corresponding panels. Scale bar = 40 µm. *N* = 5. i) Representative immunofluorescent staining of NPY (green) in the arcuate nucleus (ARC) of the hypothalamus and j) its quantitative analysis in control and HU mice. Higher magnifications of boxed areas are shown at the lower side of corresponding panels. Scale bar = 40 µm. *N* = 6. k) Representative immunofluorescent staining of NPY (green) in ARC of the hypothalamus and (l) its quantitative analysis in different treatment groups. Higher magnifications of selected areas are shown at the lower side of corresponding panels. Scale bar = 40 µm. *N* = 5. Data are presented as mean ± SEM. Statistical significance was determined by unpaired, two‐tailed Student's *t*‐test (a,b,d,j) and one‐way ANOVA with Dunnet post hoc test (f,h,l)

To examine if unloading leads to skeletal interoception, we first measured PGE2 in the hindlimbs of HU mice and found that its levels were significantly lower two weeks post hindlimbs unloading relative to the ground control mice, whereas serum PGE2 levels did not significantly change between the groups (**Figure** **1**a,b). Next, we examined if unloading resulted in skeletal interoception and changes in hypothalamic signaling. Immunofluorescent staining of TH, a marker of sympathetic activity, in the paraventricular nucleus (PVN) of hypothalamic sections, showed that TH protein levels were significantly greater in the PVN of HU mice compared to the ground control mice (Figure 1c,d). To validate that the greater sympathetic TH expression in the unloaded mice was due to decreased PGE2 in the weightless hindlimbs, we developed a hydrogel conjugate consisting of 30% F127 hydrogel with SW033291, a PGE2 degradation enzyme inhibitor, to increase local PGE2 concentrations (Figure 1e). The application of the conjugated hydrogel resulted in significantly greater PGE2 levels in the HU mice compared to control‐treated HU mice (Figure 1f). Importantly, hypothalamic TH expression in the HU mice was significantly lower upon inhibitor treatment compared to HU mice treated with the control hydrogel (Figure 1g,h), whereas no significant difference between the two treatments of control mice was seen, indicating that PGE2 elevation induced by unloading promotes hypothalamic TH expression.

NPY expression in the hypothalamus regulates energy metabolism, notably liposynthesis, to meet the energetic demands of osteoblast‐driven bone formation.^[^ ^19^ ^]^ It is noteworthy that central NPY exerts on peripheral organs through the descending neuroendocrine pathway, which is independent of the sympathetic circuit. We have also shown that the reduction of PGE2‐driven interoceptive activity significantly increases hypothalamic NPY expression by depletion of EP4 in the sensory nerve.^[^ ^19^ ^]^ Thus, we further investigated whether the expression of NPY in the hypothalamus is affected by the weightless condition. By immunostaining for NPY in hypothalamic sections, we found that NPY expression was significantly greater in the arcuate nucleus (ARC) after 2 weeks of unloading in HU mice relative to the ground control mice (Figure 1i,j). To also confirm that the greater NPY levels in the ARC are caused by down‐regulated PGE2‐driven skeletal interoception, we immunostained for NPY expression in HU mice treated with the F127/SW033291 hydrogel compared to the control hydrogel and found that the elevation of hypothalamic NPY, like TH, was blunted upon treatment compared to control treatment (Figure 1k,l). Together, these results reveal that skeletal PGE2/EP4‐driven interoceptive signaling is stimulated in the HU model via alterations in hypothalamic signaling.

### 2.2 Elevation of Hypothalamic TH Expression Induces Bone Loss

**Figure 2:**
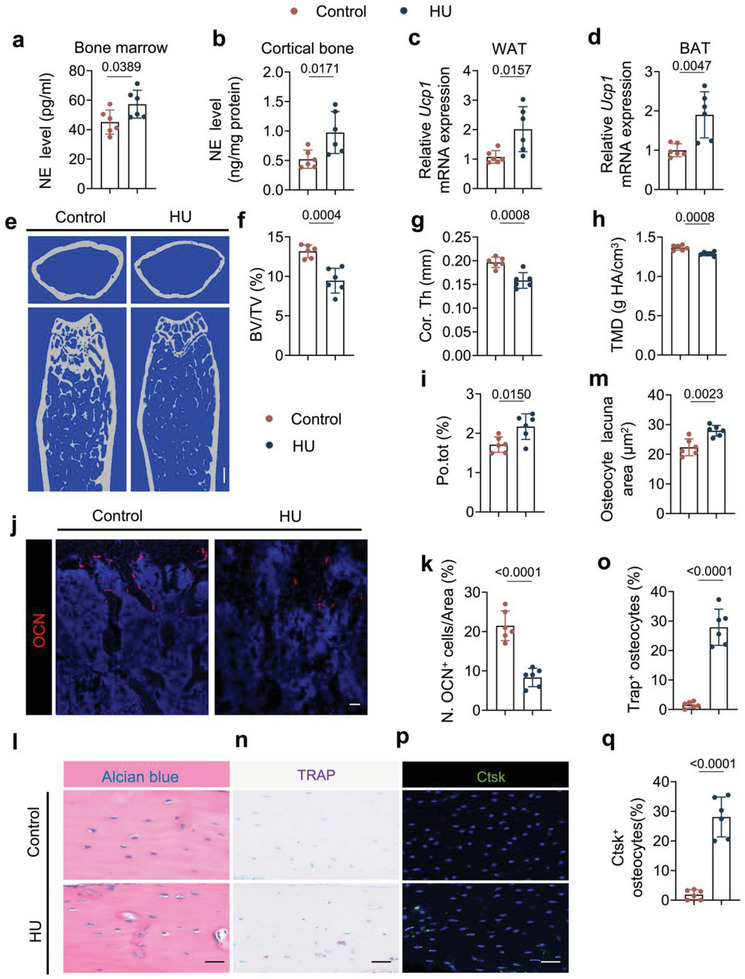
Elevated sympathetic tone induces bone loss in HU mice. a,b) ELISA analysis of norepinephrine (NE) levels in the bone marrow (a) and cortical bone (b) of control and HU mice. *N* = 6. c,d) mRNA expression of *Ucp1* by RT‐qPCR in white fat tissue (c) and brown fat tissue (d) of control and HU mice. *N* = 6. e–i) Representative micro‐computed tomography (µCT) images (e) and quantitative analysis of different bone parameters, including trabecular bone fraction (BV/TV) (f), cortical thickness (Cor.Th) (g), tissue mineral density (TMD) (h) and total porosity (Po.tot) (i) of cortical bone in control and HU mice. Scale bar = 0.25 mm. *N* = 6. j) Representative immunofluorescent staining of osteocalcin (OCN)‐positive cells (red) and k) their quantitative analysis in femoral bone marrow area of control and HU mice. *N* = 6. l) Representative Alcian blue staining and m) quantification of osteocyte lacuna area of cortical bone in control and HU mice. Scale bar = 40 µm. *N* = 6. n) Representative tartrate‐resistant acid phosphatase (TRAP) staining (purple) images at femoral mid‐shaft of cortical bone and o) quantification of TRAP^+^ osteocytes on femoral cortical bone in control and HU mice. Scale bar = 40 µm. *N* = 6. p) Representative immunofluorescent staining of Ctsk (green) at femoral mid‐shaft of cortical bone and q) quantification of Ctsk^+^ osteocytes on femoral cortical bone in control and HU mice.  Scale bar = 40 µm. *N* = 6. Data are presented as mean ± SEM. Statistical significance was determined by unpaired, two‐tailed Student's *t*‐test.

We further explored the mechanism by which elevated sympathetic activity induced bone loss in HU mice by first measuring norepinephrine (NE) levels in the hindlimb bone and found that its concentration was much higher in both the bone marrow and the cortical bone of HU mice relative to the control group (**Figure** **2**a,b), as well as in serum (Figure S1a, Supporting Information). Moreover, the expression of *Ucp1* in both the white adipose tissue (WAT) and brown adipose tissue (BAT) was also higher in the HU mice compared to the controls (Figure 2c,d), indicating that the elevated sympathetic tone in the HU mice also had effects on other peripheral organs. By microcomputed tomography (µCT) analysis we found a significantly poorer quality of the trabecular bone and cortical bone in the HU mice relative to the control mice (Figure 2e). Specifically, bone volume/tissue volume (BV/TV), cortical thickness (Cor. Th), and tissue mineral density (TMD) were lower while cortical bone total porosity (Po. Tot) was significantly greater in the HU mice compared to the ground control (Figure 2f–i). By immunofluorescent staining of femur sections, we found fewer osteocalcin (OCN)^+^ and osterix (Osx)^+^ osteoblasts in HU mice compared to ground control mice (Figure 2j,k; Figure S1b,c, Supporting Information), suggesting a compromised bone modeling in the trabecular bone. Furthermore, by histological analysis of femur cortical bone, we confirmed an enlarged osteocyte perilacunar size in HU mice compared to the control group (Figure 2l,m). Accordingly, there was an increased percentage of intracortical TRAP^+^ osteocytes and Ctsk^+^ osteocytes in the HU mice, which could explain the larger osteocyte perilacunar size and Po. tot in cortical bone of HU mice compared to ground control mice (Figure 2n–q), suggesting increased intra‐cortical bone remodeling occurs in the HU mice. Taken together, these results demonstrate that elevated sympathetic activity induces bone remodeling through skeletal interoception in HU mice.

### 2.3 The Sympathetic Antagonist Propranolol Rescues Bone Loss in Unloaded Mice

**Figure 3:**
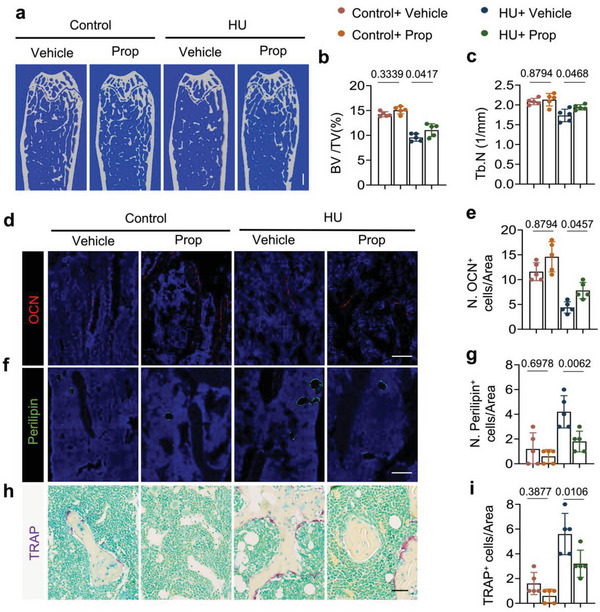
Bone loss and lipolysis activity in HU mice could be attenuated by a non‐selective β‐blocker, propranolol (Prop). Control and HU mice were injected with saline or propranolol at a dose of 0.5 mg kg^−1^ per day for 2 weeks. a–c) Representative µCT images (a) and quantitative analysis of trabecular BV/TV (b) and Trabecular number (Tb.N) (c) in different treatment groups. Scale bar = 0.5 mm. *N* = 5. d) Representative immunofluorescent staining of osteocalcin (OCN)‐positive cells (red) and e) their quantitative analysis in femoral bone marrow area of different treatment groups. Scale bar = 40 µm. *N* = 5. f) Representative immunofluorescent staining of perilipin‐positive cells (green) and (g) their quantitative analysis in femoral bone marrow area of different treatment groups. Scale bar = 40 µm. *N* = 5. h) Representative TRAP staining images and i) quantification of TRAP^+^ cells in femoral bone marrow area of different treatment groups. Scale bar = 40 µm. *N* = 5. Data are presented as mean ± SEM. Statistical significance was determined by one‐way ANOVA with the Dunnet post hoc test.

To validate the role of elevated sympathetic activity in mediating bone loss during unloading, propranolol (Prop), a non‐selective β adrenergic receptor antagonist, was intraperitoneally injected in HU mice daily for two weeks. The trabecular bone loss phenotype was rescued by Prop treatment relative to vehicle treatment in HU mice (**Figure** **3**a) as shown by µCT analysis, with BV/TV and trabecular number (Tb. N) significantly greater upon Prop treatment in HU mice compared to the vehicle control‐treated HU mice, while there is no significant difference between the two treatments of control mice (Figure 3b,c). By immunostaining of femoral sections, we found that the number of both OCN^+^ and Osx^+^ osteoblasts were significantly increased (Figure 3d,e; Figure S2a,b, Supporting Information), while the number of TRAP^+^ osteoclasts and perilipin^+^ bone marrow adipocytes were lower, in the Prop‐treated HU mice relative to vehicle‐treated HU mice (Figure 3f–i), indicating that inhibition of sympathetic tone increased osteoblastic bone formation and decreased osteoclast formation and marrow adipocyte differentiation in HU mice. These results validated the role that elevated sympathetic tone played in bone loss in HU mice.

### 2.4 Osteocyte‐Specific Deletion of *Adrb2* Attenuates Bone Loss in HU Mice

**Figure 4:**
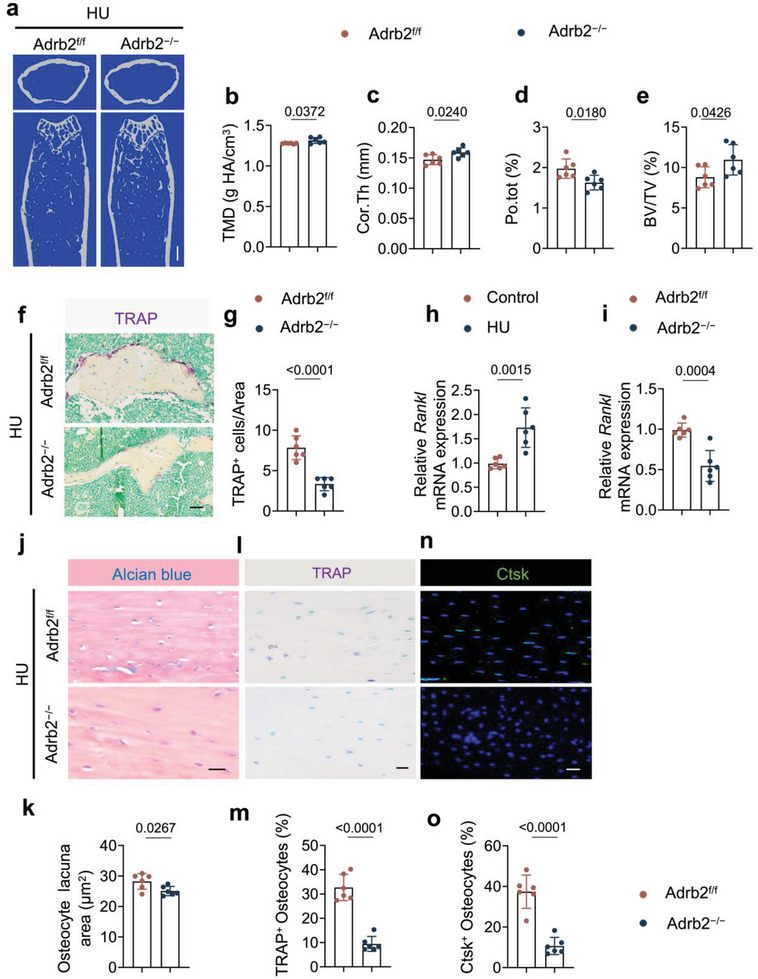
Osteocyte‐specific deletion of *Adr2b* blunts the bone loss in HU mice. a–e) Representative µCT images (a) and quantitative analysis of different bone parameters, including TMD (b), Cor.Th (c), Po.tot (d) of cortical bone and BV/TV (e) of trabecular bone of Adrb2^f/f^ and Adrb2^−/−^ HU mice. Scale bar = 0.5 mm. *N* = 6. f) Representative TRAP staining images and g) quantification of TRAP^+^ cells in femoral bone marrow area of Adrb2^f/f^ and Adrb2^−/−^ HU mice. Scale bar = 40 µm. *N* = 6. h) mRNA expression of *Rankl* by RT‐qPCR in cortical bone of control and HU mice. *N* = 6. i) mRNA expression of *Rankl* by RT‐qPCR in cortical bone of Adrb2^flox/flox^ and Adrb2^−/−^ HU mice. *N* = 6. j) Representative Alcian blue staining and (k) quantification of osteocyte lacuna area of cortical bone in Adrb2^f/f^ and Adrb2^−/−^ HU mice. Scale bar = 40 µm. *N* = 6. l) Representative TRAP staining (purple) images at femoral mid‐shaft of cortical bone and m) quantification of TRAP^+^ osteocytes on femoral cortical bone in Adrb2^f/f^ and Adrb2^−/−^ HU mice. Scale bar = 40 µm. *N* = 6. n) Representative immunofluorescent staining of Ctsk (green) at femoral mid‐shaft of cortical bone and o) quantification of Ctsk^+^ osteocytes on femoral cortical bone in Adrb2^f/f^ and Adrb2^−/−^ HU mice. Scale bar = 40 µm. *N* = 6. Data are presented as mean ± SEM. Statistical significance was determined by unpaired, two‐tailed Student's *t*‐test.

Osteocyte‐mediated perilacunar resorption has been observed in microgravity‐associated bone loss and impaired cortical bone quality.^[^ ^33^, ^34^ ^]^ To investigate if the intra‐cortical bone resorption in HU mice was driven by osteocytes, we generated *Dmp1‐cre:: Adrb2* ^flox/flox^ *(Adrb2^−/−^)* mice by crossing *Adrb2* ^flox/flox^ *(Adrb2* ^f/f^) mice with *Dmp1‐cre* mice to specifically delete *Adrb2* in osteocytes. Bone structure analysis showed that the TMD and Cor. Th were greater and that Po. tot was lower in *Adrb2* ^−/‐^ mice rather than *Adrb2* ^f/f^ mice, indicating that inhibition of sympathetic activity increased cortical quality in HU mice (**Figure** **4**a–d). Moreover, the BV/TV was also significantly recovered in *Adrb2* ^−/−^ HU mice with a lower number of TRAP^+^ osteoclasts on the trabecular surface than in *Adrb2* ^f/f^ HU mice (Figure 4e–g). The expression of *Rankl* was measured in osteocytes as Rankl is mainly expressed by these cells to induce osteoclast formation.^[^ ^35^, ^36^ ^]^ The expression of *Rankl* was indeed significantly greater after 7 days of unloading but was lower in the cortical bone of *Adrb2* ^−/‐^ unloaded mice relative to *Adrb2* ^f/f^ littermate unloaded mice (Figure 4h,i), suggesting that the regulation of *Rankl* expression acts downstream of sympathetic signaling in osteocytes. Consistent with the µCT analysis results, the osteocyte perilacuna area and the number of TRAP^+^ osteocytes and Ctsk^+^ osteocytes in the cortical bone was significantly lower in *Adrb2* ^−/−^ unloaded mice compared to their *Adrb2* ^f/f^ littermate unloaded mice (Figure 4j–o). Together, these data indicate that β‐adrenergic receptor 2 signaling in osteocytes regulates cortical bone resorption, likely via changes in *Rankl* expression in the cells.

### 2.5 Loss of Sympathetic Function Ameliorates Bone Resorption in HU Mice

**Figure 5:**
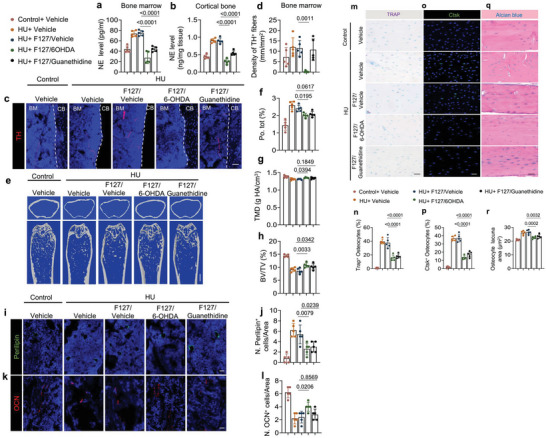
Depletion of TH‐positive sympathetic nerves or inhibition of NE release blunts bone resorption in HU mice. a,b) ELISA analysis of norepinephrine (NE) level in bone marrow (a) and cortical bone (b) of mice with different treatments. *N* = 5. c) Representative immunofluorescent staining of TH‐positive nerve fibers (red) from femoral bone marrow and d) their quantitative analysis in different treatment groups. e–h) Representative µCT images (e) and quantitative analysis of different bone parameters, including Po.tot (f), TMD (g) of cortical bone, and BV/TV (h) of trabecular bone in different treatment groups. Scale bar = 0.5 mm. *N* = 5. i) Representative immunofluorescent staining of perilipin‐positive cells (green) and j) their quantitative analysis in femoral bone marrow area of different treatment groups. Scale bar = 40 µm. *N* = 5. k) Representative immunofluorescent staining of OCN‐positive cells (red) and l) their quantitative analysis in femoral bone marrow area of different treatment groups. Scale bar = 40 µm. *N* = 5. m) Representative TRAP staining (purple) images at femoral mid‐shaft of cortical bone and n) quantification of TRAP^+^ osteocytes on femoral cortical bone in different treatment groups. Scale bar = 40 µm. *N* = 5. o) Representative immunofluorescent staining of Ctsk (green) at femoral mid‐shaft of cortical bone and p) quantification of Ctsk^+^ osteocytes on femoral cortical bone in different treatment groups. Scale bar = 40 µm. *N* = 5. q) Representative Alcian blue staining and r) quantification of osteocyte lacuna area of cortical bone in different treatment groups. Scale bar = 40 µm. *N* = 5. The conjugates of F127/Vehicle made by 30% F127 (30% w/v F127 in PBS); the conjugates of F127/6‐OHDA made by 6‐OHDA in 30% F127, the dose of 6‐OHDA was 10 mg kg^−1^ per day; the conjugates of F127/guanethidine made by guanethidine in 30% F127, the dose of guanethidine was 10 mg kg^−1^ per day. Data are presented as mean ± SEM. Statistical significance was determined by one‐way ANOVA with the Dunnet post hoc test.

To validate that increased sympathetic activity induces bone resorption during unloading, we developed a slow‐release, thermo‐responding hydrogel Pluronic F127 conjugate containing 6‐hydroxydopamine (6‐OHDA) or guanethidine to selectively deplete sympathetic nerves or to eliminate NE release, respectively. In this way, elevated TH release in response to hypothalamic signaling can be blocked at peripheral nerve terminals. F127+vehicle, F127+6‐OHDA, or F127+guanethidine was injected into one of the femurs on the 3rd day of tail suspension. The F127+6OHDA or F127+guanethidine treatment effectively blunted the rise in NE levels in both bone marrow and cortical bone relative to F127+ vehicle mice (**Figure** **5**a,b). By immunostaining femur sections, we found that there was efficient sympathetic nerve deprivation in the F127+6OHDA‐treated group (Figure 5c,d). Importantly, the loss of bone in unloaded mice was significantly prevented by treatments of F127+6OHDA or F127+guanethidine (Figure 5e). Specifically, the percentages of Po.tot were significantly lower, along with an increase in TMD in the F127+6OHDA‐treated group relative to the F127+vehicle‐treated mice. Moreover, the BV/TV in unloaded mice was significantly recovered upon F127+6OHDA or F127+guanethidine treatment relative to the F127+vehicle‐treated mice (Figure 5f–h). By immunostaining for perilipin, OCN, and Osx, we found there was significant decrease of perilipin^+^ adipocytes and increase of OCN^+^ and Osx^+^ osteoblasts in the unloaded mice treated with F127+6OHDA compared to the unloaded mice treated with F127+vehicle, whereas there were only significant fewer perilipin^+^ adipocytes in the unloaded mice treated with F127+guanethidine relative to the unloaded mice treated with F127+vehicle (Figure 5i–l; Figure S3a,b, Supporting Information). By TRAP staining and immunostaining for Ctsk in femur sections we found that there were fewer TRAP^+^ osteocytes and Ctsk^+^ osteocytes in F127+6OHDA‐ or F127+guanethidine‐treated unloaded mice relative to the vehicle‐treated unloaded group (Figure 5m–p). And by Alcian blue staining of femur sections, we found that the greater osteocyte lacuna area in cortical bone of unloaded mice was blunted by treatment with F127+6OHDA or F127+guanethidine (Figure 5q,r). Together, these results suggest that the elimination of TH^+^ sympathetic nerves or reduction of NE secretion inhibits both trabecular and cortical bone resorption mediated by osteocytes and osteoclasts. Further, the finding that inhibition of NE release only partly rescued bone volume suggests that the increase of hypothalamic NPY in unloading is a neuroendocrine regulation for energy metabolism.

### 2.6 Elevation of Hypothalamic NPY Regulates Fat Metabolism During Unloading

**Figure 6:**
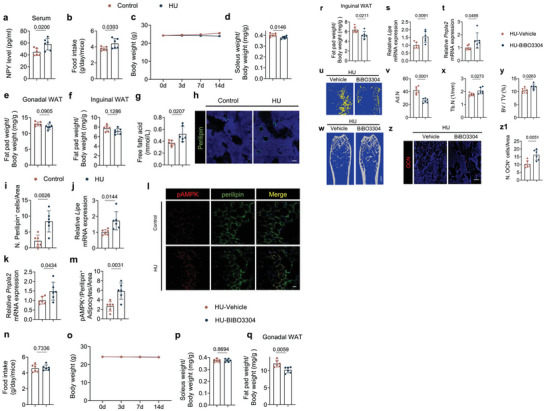
HU mice display greater NPY expression and inhibition of NPY Y1R blunts the negative effects on bone and fat metabolism of unloading. a) ELISA analysis of NPY level in serum of control and HU mice. *N* = 6. b) Quantitative analysis of food intake for control and HU mice. *N* = 6. c) Quantitative analysis of body weight for control and HU mice. *N* = 6. d–f) Quantitative analysis of the weight of the soleus (d), the gonadal (e), and inguinal fat pads (f) isolated from control and HU mice. *N* = 6. g) ELISA analysis of free fatty acid level in serum from control and HU mice. *N* = 6. h) Representative immunofluorescent staining of perilipin‐positive cells (green) and their quantitative analysis i) in femoral bone marrow area of control and HU mice. Scale bar = 40 µm. *N* = 6. j,k) mRNA expression of *Lipe* (j) and *Pnpla2* (k) by RT‐qPCR in white adipose tissues of control and HU mice. *N* = 6. l,m) Representative co‐immunofluorescent staining of pAMPK (red) and perilipin (green) from gonadal white adipose tissues (l) and quantitative analysis of pAMPK^+^/Perilipin^+^ adipocytes per area (m) in control and HU mice. Scale bar = 40 µm. *N* = 6. n) Quantitative analysis of food intake for HU mice treated with vehicle and BIBO3304 (1 mg kg^−1^ per day) every day for 2 weeks. *N* = 6. o) Quantitative analysis of body weight for HU mice treated with vehicle and BIBO3304. *N* = 6. p–r) Quantitative analysis of the weight of the soleus (p) and gonadal fat pads (q), and the inguinal fat pads (r) isolated from HU mice treated with vehicle and BIBO3304. *N* = 6. s,t) mRNA expression of *Lipe* (s) and *Pnpla2* (t) by RT‐qPCR in white adipose tissues of HU mice treated with vehicle and BIBO3304. *N* = 6. u,v) Representative µCT‐detected OsO_4_‐stained images of decalcified femurs (u) and quantitative analysis of the number of adipocytes (Ad.N) (v) in HU mice treated with vehicle and BIBO3304. *N* = 6. Scale bar = 0.5 mm. w–y) Representative µCT images (w) and quantitative analysis of different bone parameters, including trabecular number (Tb.N) (x) and trabecular BV/TV (y) in HU mice treated with vehicle and BIBO3304, respectively. Scale bar = 1 mm. *N* = 6. z,z1) Representative immunofluorescent staining of OCN (red) (z) and quantification of OCN‐positive cells (z1) in femoral bone marrow area of HU mice treated with vehicle and BIBO3304, respectively. *N* = 6. Scale bar = 40 µm. Data are presented as mean ± SEM. Statistical significance was determined by unpaired, two‐tailed Student's *t*‐test.

NPY is a neuroendocrine factor that controls appetite, lipogenesis, and the balance of bone and fat metabolism.^[^ ^37^, ^38^, ^39^ ^]^ We, therefore, examined if hypothalamic NPY regulates fat and bone metabolism to facilitate interoceptive TH‐regulated bone remodeling using unloaded mice. First, we examined serum NPY concentration by ELISA and found that NPY levels were significantly higher in HU mice than in grounded controls (**Figure** **6**a). Food intake was also significantly greater in unloaded mice relative to the grounded controls (Figure 6b), but body weight was significantly lower in the former group relative to the controls after 2 weeks of unloading, which has been shown in different studies.^[^ ^40^, ^41^ ^]^ Like the previous report,^[^ ^42^, ^43^ ^]^ the body weight of 2 weeks unloading mice did not show a significant decrease compared to their pre‐unloading weight (Figure 6c). Moreover, the soleus muscle weight was significantly lower in unloaded mice relative to grounded mice, while the fat pad weight remained unchanged between the two groups (Figure 6d–f).

FFA catabolism is essential for bone modeling and remodeling as osteoblastic‐driven bone formation represents 20% of total bodily energy consumption.^[^ ^39^, ^44^ ^]^ Importantly, serum‐free FFA levels were significantly greater in the HU group compared to the grounded controls (Figure 6g), suggesting increased lipolysis and fat metabolism upon unloading. Indeed, the perilipin^+^ area and expression of the lipolysis markers *Lipe* and *Pnpla2* were greater in unloaded mice relative to grounded controls (Figure 6h–k). Furthermore, by co‐immunostaining of perilipin and pAMPK, we found that pAMPK^+^ adipocyte/area was significantly greater in unloaded mice than in grounded control (Figure 6l,m). Considering the elevated sympathetic tone in BAT and WAT of HU mice (Figure 2c,d), these results suggest that elevated hypothalamic NPY also affects fat metabolism during HU, however, elevated sympathetic tone caused lipolysis took predominant in energy mobilization other than NPY‐induced lipogenesis.

We next examined the effect of inhibition of NPY signaling on fat metabolism and bone resorption in unloaded mice, by intraperitoneally injection of BIBO3304, a NPY Y1 receptor antagonist.^[^ ^46^ ^]^ The food intake, body weight, and soleus muscle remained unchanged between BIBO3304‐treated and vehicle‐treated HU mice, whereas fat pad weights decreased significantly in BIBO3304‐treated HU mice (Figure 6n–r). Moreover, expression of *Lipe* and *Pnpla2* in the WAT was significantly greater (Figure 6s,t) while there were significantly fewer fat droplets detected by mCT analysis, in the unloaded mice treated with BIBO3304 relative to vehicle‐treated unloaded mice (Figure 6u,v). We next examined the bone phenotype after BIBO3304 or vehicle treatment of HU mice and found that the Tb.N and BV/TV were significantly greater compared to vehicle‐treated unloaded mice (Figure 6w–y). Moreover, by immunostaining, we found that there were fewer perilipin^+^ adipocytes and more OCN^+^ and Osx^+^ osteoblasts in BIBO3304‐treated unloaded mice compared to vehicle‐treated controls (Figure 6z,z1; Figure S4a,b, Supporting Information). Taken together, those results indicate that increased NPY expression in response to unloading affects bone and fat metabolism.

### 2.7 Reduction of NPY Expression in Hypothalamic Neurons Selectively Alters Energy Expenditure

**Figure 7:**
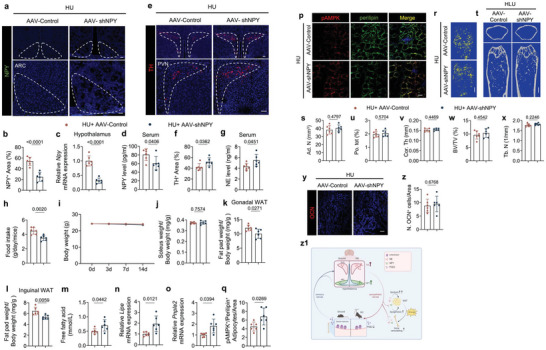
Knockdown of NPY mRNA expression in the ARC leads to alterations in energy expenditure with limited effects on TH expression and bone resorption. a) Representative immunofluorescent staining of NPY (green) in ARC of the hypothalamus and b) its quantitative analysis in HU mice treated with AAV‐Control and AAV‐shNPY, respectively. Higher magnifications of selected areas are shown at the lower side of corresponding panels. *N* = 6. Scale bar = 40 µm. c) mRNA expression of *Npy* by RT‐qPCR in the hypothalamus of HU mice treated with AAV‐Control and AAV‐shNPY. *N* = 6. d) ELISA analysis of NPY level in serum of HU mice treated with AAV‐Control and AAV‐shNPY. N = 6. e) Representative immunofluorescent staining of TH‐positive neurons and nerve fibers (red) from PVN of hypothalamus and f) their quantitative analysis in HU mice treated with AAV‐Control and AAV‐shNPY. Scale bar = 40 µm. *N* = 6. g) ELISA analysis of NE level in serum of HU mice treated with AAV‐Control and AAV‐shNPY. *N* = 6. h) Quantitative analysis of food intake for HU mice treated with AAV‐Control and AAV‐shNPY, respectively. *N* = 6. i) Quantitative analysis of body weight for HU mice treated with AAV‐Control and AAV‐shNPY, respectively. *N* = 6. j–l) Quantitative analysis of the weight of the soleus (j), the gonadal (k), and inguinal fat pads (l) isolated from HU mice treated with AAV‐Control and AAV‐shNPY, respectively. *N* = 6. m) ELISA analysis of free fatty acid level in serum from HU mice treated with AAV‐Control and AAV‐shNPY. *N* = 6. n,o) mRNA expression of *Lipe* (n) and *Pnpla2* (o) by RT‐qPCR in white adipose tissues of HU mice treated with AAV‐Control and AAV‐shNPY. *N* = 6. p) Representative co‐immunofluorescent staining and (q) quantitative analysis of pAMPK (red) and perilipin (green) from gonadal white adipose tissues of HU mice treated with AAV‐Control and AAV‐shNPY. Scale bar = 40 µm. *N* = 6. r) Representative µCT‐detected OsO_4_‐stained images of decalcified femurs and s)quantitative analysis of Ad.N in HU mice treated with AAV‐Control and AAV‐shNPY. Scale bar = 1 mm. *N* = 6. t–x) Representative µCT images (t) and quantitative analysis of different bone parameters, including Po.tot (u) and Cor.Th (v) of cortical bone, BV/TV (w), and Tb.N (x) of trabecular bone in HU mice treated with AAV‐Control and AAV‐shNPY. Scale bar = 0.5 mm. *N* = 6. y) Representative immunofluorescent staining of OCN (red) and (z) quantification of perilipin‐positive and OCN‐positive cells in femoral bone marrow area of HU mice treated with AAV‐Control and AAV‐shNPY. Scale bar = 40 µm. *N* = 6. z1) Diagram showing that decreased skeletal PGE2/EP4 ascending signal increased the expression of both sympathetic tone and NPY level in the hypothalamus as the independent descending interoceptive signal for bone and fat metabolism in the HU model. Data are presented as mean ± SEM. Statistical significance was determined by unpaired, two‐tailed Student's *t*‐test.

Although the results above show that inhibition of NPY signaling affects both bone and fat metabolism, NPY has a broad spectrum of effects, including in neurobiological and psychological processes, pain, metabolism, feeding behavior, gastrointestinal function, and other physiological systems.^[^ ^47^, ^48^ ^]^ Thus, blocking NPY signaling peripherally may not result in the same effects as preventing its hypothalamic expression. Thus, we specifically knocked down NPY mRNA expression in the ARC by intracerebroventricular (icv) injection of AAV‐mediated NPY shRNA (AAV‐shNPY), with empty AAV vector (AAV‐Control) as the control. Stereotactic injection of either AAV‐shNPY or AAV‐Control into the ARC was performed in HU mice and the brains were harvested and the hypothalamus was isolated after 2 weeks of unloading. By RT‐qPCR and immunostaining for NPY in hypothalamic sections, we found that NPY mRNA levels were successfully knocked down by AAV‐shNPY, as were serum NPY levels (**Figure** **7**a–d). Further, TH expression, as a readout of sympathetic tone, was significantly greater in the PVN as were serum NE levels in, the AAV‐shNPY‐injected group compared to the control‐injected group (Figure 7e–g). Similarly, bone marrow NE level was also significantly boosted by AAV‐shNPY‐injection in HU mice (Figure S5a, Supporting Information). The daily food intake and body weight were significantly lower in the unloaded mice injected with AAV‐shNPY relative to the AAV‐Control‐injected mice after 14 days of unloading (Figure 7h–j). The weight of both gonadal and inguinal fat pads was lower in the unloaded mice injected with AAV‐shNPY relative to the AAV‐vehicle‐injected unloaded mice, but soleus muscle weight remained unchanged between the two groups, suggesting that a selective effect of hypothalamic NPY signaling in energy metabolism during unloading (Figure 7j–l). Moreover, the serum FFA levels, as well as *Lipe* and *Pnpla2* expression, were significantly greater in the AAV‐shNPY‐injected unloaded mice compared to those injected with an AAV vehicle (Figure 7m–o). By immunostaining the WAT, we found that pAMPK^+^/perilipin^+^ adipocytes were significantly greater in number in the AAV‐shNPY treatment group compared to the control‐treated group (Figure 7p,q). However, by µCT analysis we found that the Ad.N, Po.tot, Cor. Th, BV/TV, and Tb.N did not show a statistical difference between the two groups (Figure 7r–x). Moreover, the number of OCN^+^ and Osx^+^ osteoblasts did not significantly change upon injection of AAV‐shNPY compared to the control‐injected group (Figure 7y,z; Figure S5b,c, Supporting Information). Together, the limited effect of the reduction of hypothalamic NPY on the bone indicates that the primary function of hypothalamic NPY is the regulation of energy metabolism to facilitate impaired bone formation and serve as an adaptive regulation of energy expenditure.

## 3 Discussion

Microgravity in spaceflight is the primary environmental factor that affects human health, particularly the negative impact on the skeletal system and the CNS, and thus represents a major concern for long‐duration space missions.^[^ ^47^, ^49^ ^]^ In this report, we found that unloading directly stimulates hypothalamic sympathetic tone and elevates hypothalamic NPY levels via PGE2‐driven skeletal interoception. Skeletal interoception monitors the mechanical stress on the skeletal system, as well as changes in bone density and metabolism, to allow the brain to regulate the homeostasis of the skeletal system. Under the condition of unloading or microgravity, the PGE2‐mediated ascending interoceptive signaling to the hypothalamus is decreased, and it could be anticipated that hypothalamic function is affected. Interestingly, both NPY and TH expressions in the hypothalamus were significantly higher in the HU mouse model of microgravity. As a result, bone and fat metabolic activities were altered. Importantly, the effect of unloading on hypothalamic NPY activity regulating food intake, body weight, and fat metabolism could be specifically inhibited by treatment with an NPY receptor inhibitor, whereas the effect of increased TH‐driven sympathetic activity on bone loss was selectively inhibited by treatment with an Adrb2 inhibitor or knockout of *Adrb2*. These results clearly demonstrate that unloading affects brain function, which in turn induces bone loss and increases fat metabolism (Figure 7z1). Our data uncovers for the first time the cellular and molecular mechanisms by which unloading impacts the brain. Our findings also indicate that the impact of weightlessness on bone loss is driven by alterations in the CNS.

Interoception represents the monitoring of the internal state of an organism to regulate the interactions between the brain and peripheral organs.^[^ ^22^, ^50^ ^]^ Hence, the changes in different peripheral organs under microgravity in spaceflight are likely perceived by the brain via interoception. The neural circuits of skeletal interoception respond to the altered loading signals accurately and fast. Therefore, interoception could serve as an effective platform to investigate the mechanisms by which microgravity influences the brain. Skeletal interoception is a good example of this purpose as the skeletal system is one of the most sensitive organs to microgravity. Our recent data demonstrated that bone density and metabolism are regulated by the hypothalamus through peripheral nervous innervation in the bone.^[^ ^18^, ^19^, ^21^ ^]^ Mechanical stress stimulates osteoblasts to secrete PGE2 and, in turn, PGE2‐mediated ascending interoceptive signaling depresses PVN‐driven sympathetic tone to suppress osteoblastic bone formation.^[^ ^21^ ^]^ Importantly, the hypothalamic TH^+^ sympathetic activity also determines the commitment of mesenchymal stem cells to either an osteoblast lineage or toward adipocytes.^[^ ^18^ ^]^ Under unloading conditions, the hypothalamic TH expression is elevated to induce adipocyte differentiation of mesenchymal cells, which explains the decreased osteoblast differentiation and bone loss in HU mice. In this way, the brain processes and interprets mechanical loading signals to maintain skeletal homeostasis. Therefore, microgravity‐induced bone loss likely occurs through the interaction between the brain and the skeletal system.

In addition to inducing elevated sympathetic tone, unloading also significantly increased hypothalamic NPY expression. NPY has been shown to have a broad range of regulatory functions, including neuroendocrine secretions, feeding behavior, bone and fat metabolism, circadian rhythms, neuroplasticity, and memory.^[^ ^51^, ^52^, ^53^, ^54^, ^55^ ^]^ Stress could have an impact on the central NPY expression.^[^ ^56^, ^57^ ^]^ The HU rodent model changes the physiology condition, which may increase stress. Importantly, we have demonstrated that a decrease in bone PGE2 level increases hypothalamic NPY expression. Therefore, the decrease of skeletal PGE2 level in the tail suspension mice promotes hypothalamic NPY expression through skeletal interoception. NPY is a key regulator of lipid metabolism as it increases fat synthesis and energy storage in the WAT by inhibiting BAT mobilization, whereas norepinephrine promotes lipolysis to facilitate energy mobilization by activating *Ucp1* expression in the WAT and the BAT, thereby inducing a “fight or flight” response.^[^ ^58^, ^59^, ^60^ ^]^ Different from the sensory‐sympathetic neural circuit, NPY is a neuroendocrine factor and is circulated in the blood to different tissues and organs in the balance of energy metabolism. NPY expression in the ARC is also influenced by circulating hormones, including insulin and leptin.^[^ ^61^, ^62^ ^]^ Interestingly, leptin release from the adipose tissue is stimulated by PGE2,^[^ ^63^, ^64^ ^]^ suggesting PGE2 signal could regulate hypothalamus NPY. The elevated sympathetic tone and NPY levels are offset in the adipose tissue, which could explain the non‐significant change in fat pad weight but increased serum FFA levels in HU mice. In our study, after 14 days of unloading in HU mice, food intake was significantly increased compared to ground control, but the body weight did not show a significant decrease compared to their pre‐unloading body weight. Also, no significant changes in fat mass were found between HU mice and their ground control. The observation suggests that an increase in hypothalamic NPY altered the physiological metabolic activity. Indeed, inhibition of NPY signaling in the peripheral tissue by treatment with the NPY Y1 receptor antagonist BIBO3304 rescued the loss of body weight in HU mice. Moreover, the reduction of hypothalamic NPY expression reduced the weight of fat pads but had limited effect in preventing bone loss in HU mice, validating the crucial role of hypothalamic NPY in the control of energy homeostasis to facilitate bone remodeling. Interestingly, elevated NPY in the ARC has been shown to inhibit TH expression in the PVN by activation of Y1 receptor signaling. Indeed, in our study, the down‐regulation of the central NPY level also increased the sympathetic output in HU mice. Together, these results suggest that sympathetic tone robustly increased during unloading to negatively affect the bone and fat mobilization, while the increase of NPY acts in a negative feedback manner in the hypothalamus to regulate whole‐body energy metabolism. As NPY is largely expressed in GABAergic neurons, elevated hypothalamic NPY expression in response to unloading or to microgravity likely has an impact on not only the skeletal system and metabolism but also different functions of the brain.

## 4 Experimental Section

### Mouse Strains and Constructions

C57BL/6J (WT, stock no. 000664) and *Dmp1‐Cre* (stock no. 02 3047) mouse strains were purchased from the Jackson Laboratory (Bar Harbor, ME). The *Adrb2* ^flox/flox^ mouse strain was a generous gift from Dr. Gerald Karsenty's group (Columbia University, New York, USA). To acquire the conditional knockout mice, *Dmp1‐Cre* mice were crossed with *Adrb2* ^flox/flox^ mice. The offspring were intercrossed and following genotypes were generated: *Dmp1‐Cre* (mice expressing Cre recombinase driven by the Dmp1 promoter), *Adrb2* ^flox/flox^ (mice homozygous for the *Adrb2* floxed allele and referred to as “*Adrb2* ^f/f^” in the article) and *Dmp1‐cre::Adrb2* ^flox/flox^ (mice with Adrb2 conditionally knockout in Dmp1‐positive lineage cells and referred to as “*Adrb2* ^−/−^” herein), in addition with the wild type (referred to as WT in the text). The genotypes of the mice were determined by PCR analysis of genomic DNA isolated from mouse tails with these primers: *DMP1*‐Cre forward: 5′‐TTGCCTTTCTCTCCACAGGT‐3′, *DMP1*‐Cre Reverse: 5′‐CATGTCCATCAGGTTCTTGC‐3′, *Adrb2* ^flox/flox^ forward: 5′‐AGCTGAGTGTGCAGGACGCA‐3′, and *Adrb2* ^flox/flox^ reverse: 5′‐CGCTTCGTCCCGTTCCTGAGT −3′.

All mice were kept in a temperature‐constant room with a 12:12 h light: dark cycle. All mice were housed in the animal facility of The Johns Hopkins University School of Medicine (Baltimore, MD). The Institutional Animal Care and Use Committee of Johns Hopkins University audited and approved the experimental protocol with approval number MO21M276.

The drugs and compounds used in this study are Pluronic F127 (Sigma‐Aldrich, P2443); SW033291 (S7900, Selleck, Houston, TX); Propranolol (Prop, Sigma‐Aldrich, 1 576 005); 6‐Hydroxydopamine hydrochloride (6‐OHDA, Sigma‐Aldrich, H3481); Guanethidine monosulfate (Sigma–Aldrich,1 301 801); and NPY Y1R inhibitor BIBO3304 (2412, Tocris, Minneapolis, MN). The F127‐based thermoresponsive SW033291/6‐OHDA and SW033291/Guanethidine hydrogel formulations were designed according to the previous literature.^[^ ^65^ ^]^ Dosages and time courses were noted in the corresponding text and figure legends.

### HU Animal Model

The 11–12 weeks‐old male mice were randomly separated into HU or grounded controls and hosted with one mouse per cage with available food and water. In the HU model, mice were suspended in the middle of the cage. The mice's bodies were kept at an angle of 30–40° relative to the ground with their forelimbs accessible to the cage ground while their hindlimbs were suspended in the air. By using tape and clips, mice tails were connected to the top of the lids, which promised the gesture of hindlimb unloading. The mice were checked daily to prevent hindlimbs against the cage wall or connected to the cage ground. It was guaranteed that mice had the freedom to move and had access to food and water.

Daily food intake for each mouse by was measured by weighing the remaining food in each cage and averaging over the 2 weeks experimental period. Additionally, the body weight of mice at 0, 3, 7, and 14 days was recorded during the experiment.

For biochemical analysis, whole blood samples were collected via cardiac puncture immediately after euthanasia. Serum was obtained by centrifuging the blood samples at 1500 rpm for 20 min at 4 °C and stored at −80 °C until further analysis. Femurs, brains, peripheral fat pads, and soleus muscles were also collected from the mice.

### µCT Analyses

Mice were sacrificed by inhaling isoflurane. Following a heart puncture, PBS and 4% buffered formalin subsequently were perfused. The mice femurs were dissected and fixed in 4% paraformaldehyde at 4 °C for 48 h. A high‐resolution µCT scanner (SkyScan, 1275) was used for bone parameters analysis. The scanning procedure was set as follows: 65 kV for voltage, 153 µA for current with a resolution of 6.4 µm per pixel. Reconstruction software (NRecon, v1.6, SkyScan), data analysis software (CTAn, v1.9, SkyScan), and 3D model visualization software (CTVol, v2.0, SkyScan) were used to analyze the cortical bone and the metaphyseal trabecular bone parameters of the femurs. Cross‐sectional images of the femur were reconstructed to exhibit 3D analysis of cortical bone and coronal‐sectional images for trabecular bone. The region of interest (ROI) for the trabecular bone began at proximally 0.5 mm from the distal metaphyseal growth plate and extended an additional 0.5 mm along the femur's length. The trabecular bone volume fraction (BV/TV) and trabecular number (Tb.N) were recorded from the 3D analysis data and selected to represent the trabecular bone parameters. The area between 10% of the femur's length distal to the distal metaphyseal growth plate and another 10% of the femur's length proximally was defined as the cortical bone ROI. The cortical thickness (Ct.Th), total porosity (Po.tot), and tissue mineral density of cortical bone (TMD) were selected to represent the cortical bone parameters. The tissue mineral density of cortical bone (TMD) was calibrated by Phantoms.

### OsO4 Staining and Marrow Adipose Tissue mCT Analyses

The femurs were harvested from the mice and fixed in 4% paraformaldehyde at 4 °C overnight. Then, the fixed bone samples were decalcified at 4 °C with 0.5 m ethylenediaminetetraacetic acid (EDTA) for 3 weeks. Next, the proximal ends of the decalcified femurs were removed, and the rest part of the femurs were incubated in 2% aqueous osmium tetroxide (OsO4, Sigma–Aldrich) for 2 h in the fume hood. Next, femurs were washed with phosphate‐buffered saline (PBS) for 48 h at 4 °C and scanned by using a high‐resolution µCT scanner (1172, Skyscan, Bruker Micro CT) at 45 kV and 177 µA with 6.4 µm per pixel. Quantification of marrow adipose tissue volume and distribution in bone was registered to the decalcified bone as previously described.^[^ ^18^ ^]^

### Immunofluorescence and Histomorphometry

After 3 weeks of decalcification in 0.5 m ethylenediaminetetraacetic acid (pH 7.4, Amresco), bone samples were embedded in paraffin or optimal cutting temperature (OCT) compound (Sakura Finetek, Torrance, CA). TRAP (Sigma–Aldrich) and Alcian blue staining were processed at 4 µm thick femur sections in coronal orientation by using standard protocols as used before.^[^ ^65^ ^]^ Femoral coronal‐oriented sections (10 and 40 µm thick) were collected for immunofluorescent staining by standard protocol. For preparation of brain sections, the harvested whole brain was fixed with 4% paraformaldehyde for 12 h and was dehydrated with 20% sucrose for 24 h, followed by 30% sucrose for another 24 h and sectioned in 20 µm. Immunofluorescence staining was performed using the standard protocol. Briefly, the sections were incubated with primary antibodies to rabbit Tyrosine hydroxylase (Millipore Sigma, ab152, 1:100), goat NPY (Novus, NBP1‐46535, 1:100), rabbit Cathepsin K (Abcam, ab19027, 1:100), rabbit Osteocalcin (Origene, BP710, 1:100), rabbit pAMPK (Cell Signaling Technology, 2535s, 1:100), rabbit Perilipin (Cell Signaling Technology, D1D8, 1:100), goat Perilipin (Abcam, ab60269, 1:100), rabbit Osterix (Abcam, ab22552, 1:100) at 4 °C overnight. After three times of PBS washing, the slides were then incubated with secondary antibodies conjugated with fluorochrome and DAPI (Invitrogen, Thermo Fisher Scientific H3569,1:500) at room temperature for 1 h. The images were recorded by using an Olympus DP72 microscope (Olympus Scientific Solutions Americas Inc., Waltham, MA) and a Zeiss 780 confocal microscope (Zeiss, Oberkochen, Germany). In each section, the total number of positively stained cells or signals within a randomly selected area was counted. For each group, this count was conducted in five non‐sequential sections for every mouse and calculated to obtain the average number.

### Quantitative Real‐Time Polymerase Chain Reaction (qPCR)

To extract RNA from the cortical bone, the same weight of tibias was harvested from each group of mice after perfusion with PBS. After removing the connective tissue and periosteum, the epiphysis was cut off. Bone marrow cells were flushed out with cold PBS by syringe and cortical bones were cut into 1–2 mm lengths of bone chips and stored at −80 °C before analysis.

To extract RNA from the hypothalamus, the ARC nucleus region of the hypothalamus was harvested from each group of mice after perfusion with PBS and preserved at −80 °C before analysis.

To extract RNA from WAT, the same weight of WAT in each group was harvested after perfusion with PBS and kept at −80 °C before analysis.

TRIzol reagent (Invitrogen, Thermo Fisher Scientific) was used to extract the total RNA according to the manufacturer's instructions. Then, a high‐capacity cDNA reverse transcription kit (4 374 966, Thermo Fisher Scientific) was applied to reverse transcribe the RNA into complementary DNA. QuantStudio 3 Real‐Time PCR System (Thermo Fisher Scientific) was selected to execute the RT‐qPCR with Fast SYBR Green Master Mix (4 385 610, Thermo Fisher Scientific). The 2^−ΔΔCT^ method was used to calculate the relative expression of each gene, and *Gapdh* was chosen as the internal control for normalization. The primers used for each gene were *Npy* forward: 5′‐CACGATGCT AGGTAACAA G‐3′, *Npy* reverse: 5′‐CAC ATGGAAGGGTCTTCA AG‐3′, *Lipe* forward: 5′‐CATCAACCACTGTGAGGGTAAG‐3′, *Lipe* reverse: 5′‐AAGGGAGGTGAGATGGTAACT‐3′, *Pnpla2* forward: 5′‐TAGCTAACAGTTGGGCTTCAC‐3′, *Pnpla2* reverse: 5′‐CAGAGAGAACAGAGCAGCTTAC‐3′, *Ucp1* forward: 5′‐CTGCCAGGACAGTACCCAAG‐3′, *Ucp1* reverse: 5′‐TCAGCTGTTCAAAGCACACA‐3′, *Rankl* forward: 5′‐ATCGGGAAGCGTACCTACAG‐3′, *Rankl* forward: 5′‐GTGCTCCCTCCTTTCATCAG‐3′, *Gapdh* forward: 5′‐GGGTGTGAACCACGAGAAAT‐3′, and *Gapdh* reverse: 5′‐CCTTCCACAATGCCAAAGTT‐3′

### Cell Lines

AAV‐293 cells were used for viral preparation. Cells were cultured in a DMEM growth medium with 4.5 g L^−1^ glucose, 110 mg L^−1^ sodium pyruvate, and 4 mm L‐glutamin. An additional 10% (v/v) heat‐inactivated fetal bovine serum was supplemented during cell culture.

### ELISA

Mice were sacrificed from each group and their tibias were dissected. Soft tissue was removed through scraping, and both epiphyses were cut off. Bone marrow cells were centrifuged for 15 min at 3000 r.p.m. at 4 °C to obtain bone marrow supernatants. Isolated supernatants were stored at −80 °C until the ELISA assay. The cortical bone was cut into 1–2 mm and both isolated supernatants and bone pieces were kept at −80 °C until the ELISA assay. The serum supernatants were harvested by centrifuging for 20 min at 3000 r.p.m. at 4 °C and stored at −80 °C until the experiment. Expression of NE was measured by a high‐sensitivity NE ELISA kit (Eagle Biosciences), PGE2 level was determined by a PGE2 ELISA kit (Cayman Chemical), and NPY level was determined by NPY ELISA kit (NOVUS). The same weight of bone chips or volume of supernatants was used from each sample to quantify their protein concentrations and to normalize NE measurements. The manufacturer's protocol was followed for each ELISA kit.

### AAV‐Mediated Vector Preparation

An AAV‐mediated vector was prepared according to the protocol published before.^[^ ^64^ ^]^ In detail, the mouse shNPY with U6 promotor (Origene) was cloned into the pAAV‐IRES‐hrGFP vector to make a recombinant plasmid of pAAV‐shNPY and pAAV‐shControl (Agilent Technologies). Then, pAAVshNPY (or pAAV shCTL), pHelper (carrying adenovirus‐derived genes), and pAAV‐RC (carrying AAV‐2 replication and capsid genes), those three plasmids were co‐transfected into cells according to the manufacturer's protocol (Agilent Technologies). Cells were harvested after 72 h of transfection, and the recombinant viral vector AAV‐shNPY (or AAV‐shControl) was collected by centrifuge at 10 000 g for 10 min according to the manufacturer's protocols. Quantitative PCR was used to determine the virus titers and 1 × 10^9^ particles/site was used for each virus injection.

### Stereotaxic Injection of AAV Vector

Mice stereotactic injection was performed according to the protocol.^[^ ^64^, ^65^ ^]^ In detail, mice were randomly divided into two groups and placed in a stereotaxic frame after anesthetized. The skull was exposed after disinfection and the bregma was located. 30G syringe was used to drill a hole in the skull and a 5 µL micro syringe (Hamilton) was used to execute administration. The coordinate is 1.6 mm posterior from bregma, along to the midline at a depth of 2.0 mm. AAV‐Control or AAV‐shNPY was delivered into the third ventricle at a volume of 1 µL for 1 min, and the needle was withdrawn 3 min after the end of the injection. After 5 days post recovery, animals were used for experiments.

### Statistical Analysis

All error bars exhibited standard deviation (SD). Data were presented as mean ± SD. Unpaired, two‐tailed Student's *t*‐tests were used for comparisons between two groups and one‐way analysis of variance (ANOVA) with Dunnett post hoc test or two‐way analysis of variance (ANOVA) with Tuckey post hoc test multiple comparisons herein. All data demonstrated a normal distribution and similar variation between groups. All inclusion/exclusion criteria were predetermined, and no samples or animals were excluded. The sample size was not determined using any statistical method. The experiments were all randomized. During the experiments and outcome evaluation, the investigators were not blinded to allocation.

## Conflict of Interest

The authors declare no conflict of interest.

## Supporting information

Supporting Information

## Data Availability

The data that support the findings of this study are available from the corresponding author upon reasonable request.
