# Supplementary material for: Unloading‐Induced Skeletal Interoception Alters Hypothalamic Signaling to Promote Bone Loss and Fat Metabolism
Source: Adv Sci (Weinh). 2023 Oct 25;10(35):2305042. doi: 10.1002/advs.202305042 (PMC10724445; doi:10.1002/advs.202305042)
Supplement: Supplementary file 1 — Supporting Information [file ADVS-10-2305042-s001.pdf]

ADVANCED SCIENCE
Open Access

Supporting Information

for *Adv. Sci.*, DOI 10.1002/advs.202305042

Unloading-Induced Skeletal Interoception Alters Hypothalamic Signaling to Promote Bone Loss and Fat Metabolism

*Qiaoyue Guo, Ningrong Chen, Kalp Patel, Mei Wan, Junying Zheng and Xu Cao**

Supplementary data

Fig. S1

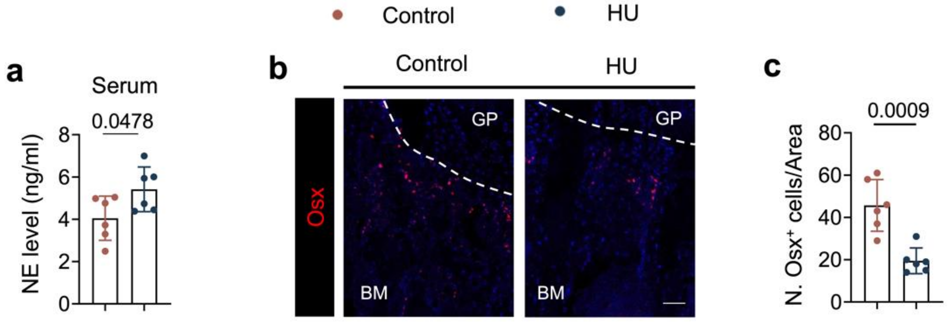

Fig. S1: Sympathetic tone was increased with inhibition of Osx positive osteoblasts in bone marrow during HU.
(a) Enzyme-linked immunosorbent assay (ELISA) analysis of NE level in serum of control and HU mice. N=6. (b) Representative immunofluorescent staining of Osterix (Osx)-positive cells (red) and (c) their quantitative analysis in femoral bone marrow area of control and HU mice. Scale bar=40μm. N=6.

Fig. S2

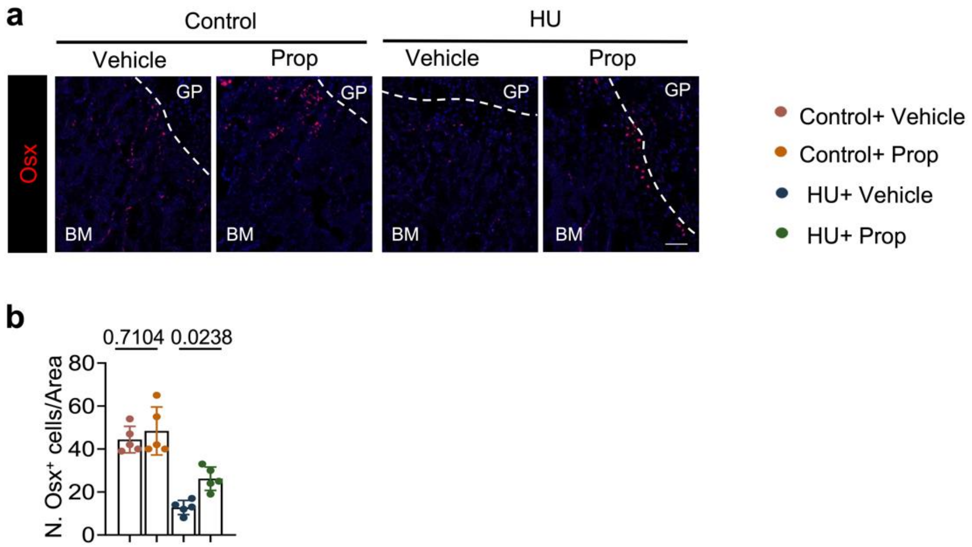

Fig. S2: The number of Osx positive osteoblasts could be attenuated by propranolol (Prop) in HU mice.
(a) Representative immunofluorescent staining of Osx^+ cells (red) and (b) their quantitative analysis in femoral bone marrow area of different treatment group. GP for growth plate and BM for bone marrow. Scale bar= 40μM. N=5.

Fig. S3

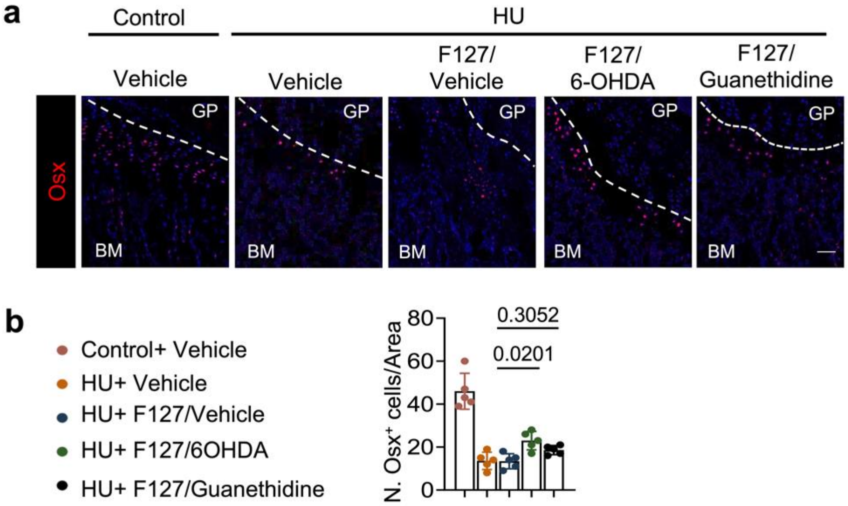

Fig. S3: The number of Osx positive osteoblasts in bone marrow could recovered by depletion of TH-positive sympathetic nerves in HU mice.

(a) Representative immunofluorescent staining of Osx$^+$ cells (red) and (b) their quantitative analysis in femoral bone marrow area of each treatment groups. GP for growth plate and BM for bone marrow. Scale bar=40μm, N=5.

Fig. S4

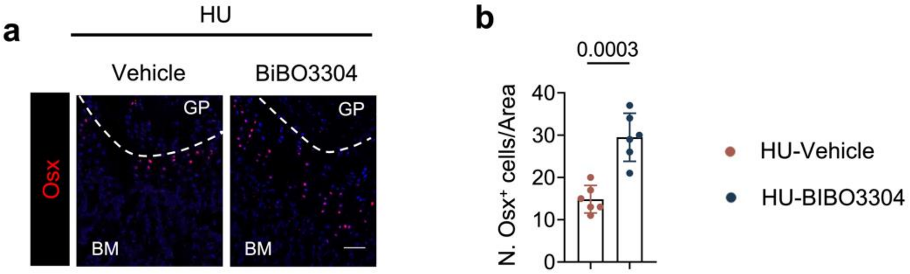

Fig. S4: The number of Osx positive osteoblasts in bone marrow could be rescued by treatment of NPY Y1R inhibitor in HU mice.

(a)Representative immunofluorescent staining of Osx$^+$ cells (red) and (b) their quantitative analysis in femoral bone marrow area of vehicle and BIBO3304 treated HU mice. Scale bar=40μm, N= 6.

Fig. S5

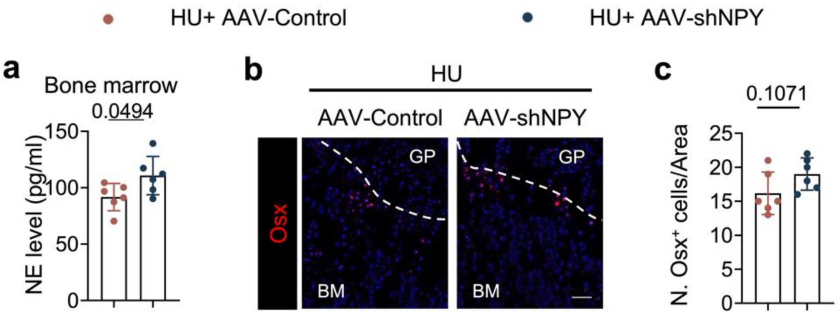

Fig. S5 Knockdown of NPY mRNA expression in the ARC increased sympathetic activity with limited effects

on Osx positive osteoblasts in bone marrow of HU mice.

(a) ELISA evaluation of NE level in serum of AAV-Control and AAV-shNPY treated HU mice. (b)Representative immunofluorescent staining of osterix (Osx)-positive cells (red) and (b) their quantitative analysis in femoral bone marrow area of AAV-Control and AAV-shNPY treated HU mice. Scale bar=40μm, N= 6.
